# Peripheral endocrine–nutritional machine learning signature predicts short-term response to neoadjuvant immunochemotherapy in locally advanced gastric cancer

**DOI:** 10.3389/fimmu.2026.1907116

**Published:** 2026-08-12

**Authors:** Fang Li, Zefan Mu, Honghai Guo, Tao Zheng, Peigang Yang, Zhiqiang Wang, Yanlong Shi, Wen Xu, Yuan Tian, Wenqian Ma, Qun Zhao

**Affiliations:** 1 Department of Pathology, The Fourth Hospital of Hebei Medical University, Shijiazhuang, China; 2 The Third Department of Surgery, The Fourth Hospital of Hebei Medical University, Shijiazhuang, China; 3 Hebei Key Laboratory of Precision Diagnosis and Comprehensive Treatment of Gastric Cancer, Shijiazhuang, China; 4 Big Data Analysis and Mining Application for Precise Diagnosis and Treatment of Gastric Cancer Hebei Provincial Engineering Research Center, Shijiazhuang, China; 5 Department of General Surgery, Shijiazhuang People’s Hospital, Shijiazhuang, China; 6 Department of Gastrointestinal Surgery, The Fifth Affiliated Hospital of Anhui Medical University, Hefei, China; 7 Department of Medical Record, The First College of Clinical Medical Science, China Three Gorges University and Yichang Central People’s Hospital, Yichang, China; 8 Department of Endoscopy, The Fourth Hospital of Hebei Medical University, Shijiazhuang, China

**Keywords:** gastric cancer, host endocrine-nutritional state, machine learning, major pathological response, neoadjuvant immunochemotherapy, risk stratification

## Abstract

**Background:**

Major pathological response (MPR) to neoadjuvant PD-1 inhibitor plus chemotherapy in locally advanced gastric cancer (LAGC) varies widely and is incompletely explained by tumor-side biomarkers. Because checkpoint inhibitors act within a host environment shaped by endocrine, nutritional, and inflammatory state, we developed and validated the Endocrine–Nutritional Immunotherapy response score (ENI-Score) from routine pretreatment peripheral-blood markers to predict short-term response and early recurrence.

**Methods:**

We retrospectively analyzed 738 patients with LAGC or gastroesophageal junction adenocarcinoma who received neoadjuvant PD-1 inhibitor plus platinum-based chemotherapy and radical gastrectomy at four hospitals, partitioned into training (n=335), internal (n=114), and external (n=289) validation cohorts. Ten machine-learning algorithms were benchmarked, and a prespecified, domain-balanced five-marker panel comprising 8 AM cortisol, prognostic nutritional index (PNI), prealbumin, neutrophil-to-lymphocyte ratio (NLR), and C-reactive protein-to-albumin ratio (CAR) was carried forward in a regularized logistic model and rescaled to the ENI-Score (0 -100). Analyses included SHAP, tertile stratification, nested AUC comparison (DeLong), calibration, decision-curve analysis, and Kaplan–Meier survival analysis; the primary endpoint was MPR (residual viable tumor ≤10%).

**Results:**

The overall MPR rate was 40.9% (302/738). All five markers differed significantly between MPR and non-MPR patients in a biologically coherent direction consistent across all three cohorts (each P<0.001). Regularized logistic regression achieved the highest, most stable external-validation AUC among the ten algorithms (training 0.873, internal 0.816, external 0.830, and five-fold cross-validated 0.862), whereas tree-based ensembles overfit (training AUC up to 0.99, external ≤0.81). ENI-Score tertiles separated MPR rates steeply (Low 10.4%, Intermediate 38.2%, High 79.1%; P-trend<0.001), with an adjusted High-benefit versus Low-benefit odds ratio of 35.3 (95% CI 20.6-60.5) and a per 10-point adjusted OR of 1.72 (95% CI 1.59-1.86, P<0.001). The ENI-Score reached an overall AUC of 0.842 and significantly augmented clinical staging (0.656 to 0.864; DeLong P<0.001). Higher ENI-Score was associated with longer recurrence-free survival (RFS; log-rank P<0.001; adjusted HR per 10 points 0.78).

**Conclusions:**

Built entirely from five low-cost routine peripheral-blood markers, the ENI-Score accurately and reproducibly stratifies MPR after neoadjuvant immunochemotherapy in LAGC, augments tumor-centered clinical staging, and is associated with RFS. It provides an inexpensive, interpretable host-state adjunct to tumor-side biomarkers that warrants prospective validation.

## Introduction

Gastric cancer is the fifth most common malignancy and a leading cause of cancer death worldwide, with China contributing a disproportionate share of incident cases and deaths each year (1, 2). Because symptoms appear late, the majority of Chinese patients are diagnosed with locally advanced disease, for which contemporary guidelines recommend neoadjuvant systemic therapy followed by radical gastrectomy with D2 lymphadenectomy (3, 4). Perioperative triplet chemotherapy improved survival over older doublets in Western populations (5), and perioperative or adjuvant oxaliplatin-based regimens are widely used across Asia (6). The most consequential recent change has been the addition of programmed cell death-1 (PD-1) or programmed death-ligand 1 (PD-L1) inhibitors to a fluoropyrimidine-platinum backbone. In the metastatic setting, this combination prolonged survival in CheckMate 649, ORIENT-16, and KEYNOTE-859 (7–9), and the strategy has now moved into the curative-intent perioperative space: KEYNOTE-585 improved pathological complete response without a significant event-free survival benefit, whereas MATTERHORN demonstrated a significant event-free survival benefit for perioperative durvalumab plus FLOT (10, 11). Pathological response after neoadjuvant therapy, in particular major pathological response (MPR) and pathological complete response (pCR), has therefore become a clinically meaningful early endpoint that is associated with long-term outcome and is increasingly used to guide perioperative decision-making. Identifying, before treatment, which patients are likely to achieve a deep pathological response carries direct clinical significance: it could spare probable non-responders from weeks of ineffective therapy and the attendant toxicity and surgical delay, support earlier escalation or a change of strategy, and inform shared decision-making, all of which argue for accurate, inexpensive, and timely predictive tools.

Despite these advances, the depth of pathological response remains strikingly heterogeneous between patients who receive nominally identical regimens. A sizeable fraction achieve MPR or pCR, yet many derive limited benefit and are exposed to toxicity and to a delay in definitive surgery without a commensurate oncological gain. Tumor-side biomarkers explain part of this variability. PD-L1 combined positive score (CPS), mismatch-repair (MMR) or microsatellite-instability (MSI) status, Epstein–Barr virus (EBV) positivity, and the broader molecular subtypes derived from comprehensive genomic characterization each enrich for response, and patients with MSI-high or EBV-positive tumors can show exceptional sensitivity to PD-1 blockade (12–14). However, the survival benefit of immunochemotherapy is concentrated in the CPS ≥5 subgroup, MSI-high and EBV-positive tumors together account for well under one-fifth of cases, and a substantial proportion of biomarker-enriched patients still fail to respond (7, 8, 13). Tumor-centered tests are also tissue-dependent, are not uniformly available in real time, and capture only one side of the response equation. Markers that are inexpensive, reproducible, available before treatment, and that index a complementary axis of biology are therefore of considerable practical value.

Immune checkpoint inhibitors do not act in isolation; their efficacy is conditioned by the systemic host environment, spanning endocrine, nutritional, and inflammatory state (15, 16). Endogenous glucocorticoid signaling, for which morning cortisol is a convenient peripheral surrogate, transactivates checkpoint receptors and drives CD8 T-cell dysfunction in the tumor microenvironment, and active glucocorticoid signaling has been linked to failure of checkpoint blockade in preclinical models and patients (15–17). Depleted nutritional reserve, reflected by hypoalbuminemia, low prealbumin, and a low prognostic nutritional index (PNI), limits the capacity to mount and sustain an effector response, and pretreatment PNI predicts immunotherapy outcome across tumor types (18, 19). A myeloid-skewed, systemically inflamed state, indexed by an elevated neutrophil-to-lymphocyte ratio (NLR), C-reactive protein-to-albumin ratio (CAR), and systemic immune-inflammation index, has repeatedly been associated with inferior immunotherapy efficacy and survival (18, 20). Most prior studies, however, examined a single marker or a single domain in isolation, and integrated, interpretable tools that combine the endocrine, nutritional, and inflammatory axes for neoadjuvant gastric cancer remain scarce. In parallel, our group and others have shown that multimodal artificial-intelligence models built from imaging, pathology, and transcriptomic data can non-invasively predict occult peritoneal metastasis, lavage-cytology positivity, and postoperative recurrence in gastric cancer (21–28), underscoring both the promise of data-driven risk stratification and the fact that the low-cost peripheral host-state axis has been comparatively underexploited for predicting neoadjuvant response.

Against this background, we designed a multicenter study of 738 patients with locally advanced gastric or gastroesophageal junction adenocarcinoma treated with neoadjuvant PD-1 inhibitor plus chemotherapy. We compared a comprehensive panel of pretreatment host-state markers between MPR and non-MPR patients, benchmarked ten commonly used machine-learning algorithms, and assembled a domain-balanced peripheral-blood signature, the Endocrine–Nutritional Immunotherapy response score (ENI-Score). We then evaluated whether the ENI-Score stratifies MPR probability, how it ranks against and adds to a clinical-staging baseline, how individual markers contribute through interpretable attribution, and whether the score carries prognostic signal for early recurrence, with the explicit goal of characterizing the magnitude and the limits of the host-state contribution rather than overstating it.

## Materials and methods

### Study design and patient population

**Figure 1:**
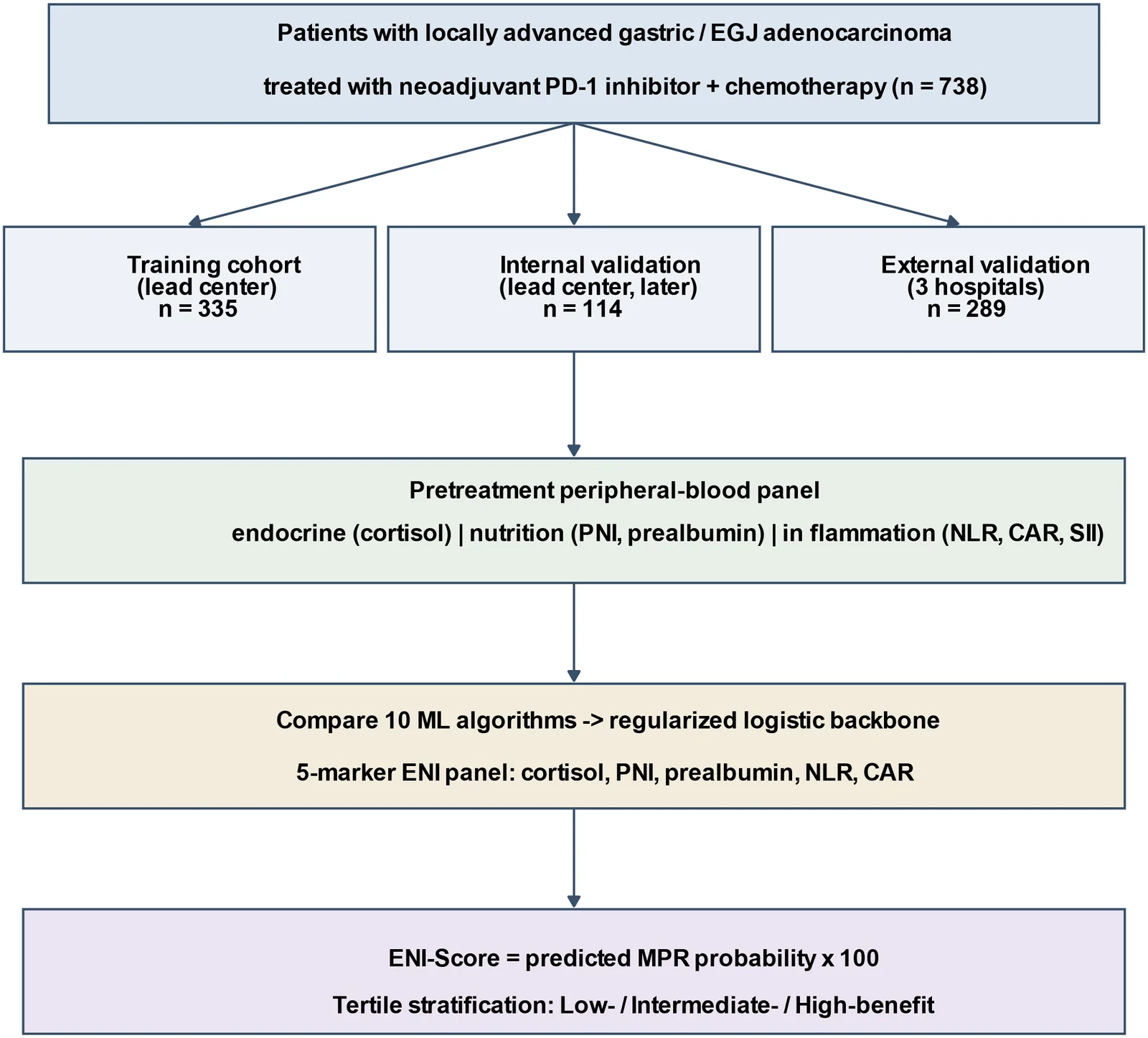
Study design and ENI-score workflow, from multicenter cohort allocation through pretreatment marker collection, machine-learning model comparison, and tertile-based clinical stratification.

This multicenter retrospective study enrolled consecutive patients treated between 2020 and 2023 at four hospitals: The Fourth Hospital of Hebei Medical University served as the lead center, and Shijiazhuang People’s Hospital, The Fifth Affiliated Hospital of Anhui Medical University, and Yichang Central People’s Hospital served as external centers. The protocol was approved by the Ethics Committee of the Fourth Hospital of Hebei Medical University and by the institutional review boards of each participating center; given the retrospective design and the use of de-identified data, the requirement for written informed consent was waived in accordance with national legislation. The study was conducted in accordance with the Declaration of Helsinki and is reported in line with the Transparent Reporting of a multivariable prediction model for Individual Prognosis Or Diagnosis (TRIPOD) statement. The screening, allocation, and ENI-Score construction workflow is summarized in **Figure 1**.

Eligible patients had histologically confirmed gastric or gastroesophageal junction adenocarcinoma; locally advanced disease (cT3-4a/b and/or clinically node-positive) staged by the 8th edition AJCC/UICC TNM system using contrast-enhanced computed tomography and endoscopy; a neoadjuvant regimen combining a PD-1/PD-L1 inhibitor with platinum-based chemotherapy for at least two preoperative cycles; subsequent radical gastrectomy with D2 lymphadenectomy; a complete pretreatment panel of endocrine, nutritional, micronutrient, and inflammatory markers obtained within a fixed window before the first cycle; and an interpretable postoperative pathological response assessment. Patients were excluded for distant metastasis at diagnosis, failure to complete planned neoadjuvant therapy or surgery, active infection, autoimmune disease or systemic corticosteroid therapy within the sampling window, severe missingness of key laboratory variables, or a non-evaluable pathological specimen. Cohort allocation followed enrollment center and a non-overlapping time window: 335 earlier lead-center patients formed the training cohort, 114 later lead-center patients formed the internal validation cohort, and 289 patients from the three external centers formed the external validation cohort. After the screening and exclusion sequence (exclusion of distant metastasis, incomplete neoadjuvant therapy, no radical surgery, incomplete pretreatment laboratory data, and non-evaluable pathology), the final analytic population comprised 738 patients. Markers were required to be complete by design, and patients with severe missingness of key laboratory variables were excluded rather than imputed, so no imputation was applied to the analytic cohort; this conservative handling is acknowledged as a potential source of selection bias in the Discussion. Baseline characteristics by cohort are summarized in **Table 1**.

**Table 1:** Baseline characteristics by cohort.

Variable	Training (n=335)	Internal (n=114)	External (n=289)	P
Age, years	61.0 (56.0-67.0)	62.0 (56.2-67.0)	62.0 (56.0-68.0)	0.909
Male sex	219 (65.4)	82 (71.9)	189 (65.4)	0.396
BMI, kg/m²	22.6 (20.6-24.6)	22.4 (20.4-24.1)	22.7 (21.0-24.6)	0.506
ECOG PS ≥1	141 (42.1)	52 (45.6)	122 (42.2)	0.789
EGJ/upper-third	40 (11.9)	11 (9.6)	45 (15.6)	0.207
Clinical T4	152 (45.4)	55 (48.2)	145 (50.2)	0.485
Clinical N2/N3	173 (51.6)	61 (53.5)	163 (56.4)	0.492
PD-L1 CPS ≥5	205 (61.2)	63 (55.3)	174 (60.2)	0.531
dMMR/MSI-H	16 (4.8)	8 (7.0)	30 (10.4)	0.027
Cortisol, µg/dL	13.3 (11.4-15.0)	13.5 (12.4-15.3)	13.7 (12.1-15.4)	0.155
PNI	48.5 (45.6-51.6)	47.7 (45.2-50.7)	48.0 (44.9-50.9)	0.186
Prealbumin, mg/L	236 (208-267)	232 (208-261)	236 (208-260)	0.599
NLR	2.4 (1.9-3.2)	2.6 (2.0-3.4)	2.6 (2.0-3.3)	0.145
CAR	0.13 (0.07-0.21)	0.14 (0.08-0.22)	0.13 (0.07-0.21)	0.338
ENI-Score	37.0 (13.1-67.3)	26.6 (12.4-56.0)	30.3 (10.6-59.7)	0.196
MPR	136 (40.6)	48 (42.1)	118 (40.8)	0.960
pCR	44 (13.1)	18 (15.8)	50 (17.3)	0.344
R0 resection	314 (93.7)	106 (93.0)	264 (91.3)	0.518

Continuous variables are median (IQR); categorical variables are n (%). P values are from Kruskal–Wallis or χ² tests across cohorts. ECOG PS, Eastern Cooperative Oncology Group performance status; EGJ, gastroesophageal junction; PNI, prognostic nutritional index; NLR, neutrophil-to-lymphocyte ratio; CAR, C-reactive protein-to-albumin ratio; MPR, major pathological response; pCR, pathological complete response.

### Pretreatment host-state markers and composite indices

All peripheral-blood markers were obtained in the fasting state before the first neoadjuvant cycle and were measured by standardized clinical assays at each center. Candidate host-state markers spanned four predefined domains: endocrine (8 AM serum cortisol, thyroid-stimulating hormone, free triiodothyronine, free thyroxine), nutritional (albumin, prealbumin, total protein, hemoglobin, body mass index, PNI), micronutrient and iron (25-hydroxy vitamin D, serum iron, ferritin, transferrin), and inflammatory (white-blood-cell count, neutrophil, lymphocyte, monocyte, platelet, C-reactive protein [CRP], lactate dehydrogenase, NLR, platelet-to-lymphocyte ratio, lymphocyte-to-monocyte ratio, systemic immune-inflammation index [SII], systemic inflammation response index, CAR). Composite indices were calculated as PNI = albumin (g/L) + 5 × lymphocyte count (10^9^/L) (29); NLR = neutrophil/lymphocyte; CAR = CRP/albumin; and SII = platelet × neutrophil/lymphocyte. All feature handling, including standardization, was confined to the training cohort to prevent information leakage; continuous markers were transformed to z-scores using training-cohort means and standard deviations, which were then applied unchanged to the validation cohorts.

### ENI-score development

The five markers were selected before model training on the basis of three prespecified criteria: biological representation of the endocrine, nutritional, and inflammatory axes; routine clinical availability and low cost; and limited redundancy with an acceptable degree of inter-marker correlation. The full list of candidate markers considered, with the rationale for inclusion or exclusion, is provided in **Supplementary Table 1**. Accordingly, we prespecified a compact, domain-balanced five-marker panel before model fitting: 8 AM cortisol (endocrine), PNI and prealbumin (nutritional), and NLR and CAR (inflammatory). This panel was carried forward in a regularized (L2-penalized) logistic regression model, with the penalty strength selected by five-fold cross-validation in the training cohort. The model-estimated probability of MPR was multiplied by 100 to yield the ENI-Score, ranging from 0 to 100, with higher values indicating a higher predicted probability of benefit. Tertile cutoffs derived from the training-cohort score distribution defined Low-benefit, Intermediate-benefit, and High-benefit groups and were applied without modification to both validation cohorts. To avoid in-sample optimism, training-cohort ENI-Scores were generated by five-fold out-of-fold prediction, whereas validation-cohort scores used the model refitted on the full training cohort. The complete ENI-Score equation, including the intercept, the standardized regression coefficients, the training-cohort means and standard deviations used for z-score transformation, and the tertile cut-points, is reported in **Supplementary Table 2** so that the score can be recomputed externally; markers were z-scored using training-cohort statistics, and no missing-value imputation was required because complete marker data were an inclusion criterion.

### Outcome definitions

The primary endpoint was MPR, defined as ≤10% residual viable tumor in the resected primary specimen on pathological review performed with standardized criteria at each center and central adjudication when necessary, with reviewers blinded to ENI-Score and clinical outcome; tumor regression was graded using an established gastric regression scheme (30). The scheme applied was the Becker tumor regression grading system, and every specimen near the major-pathological-response threshold or with any inter-reader uncertainty underwent independent central re-review by two gastrointestinal pathologists at the lead center with disagreements resolved by consensus; all pathological reviewers were blinded to the pretreatment peripheral-blood markers as well as to the ENI-Score and clinical outcome. Secondary endpoints included pCR (no residual viable tumor in primary and nodes), objective response rate (ORR) and disease control rate (DCR) by RECIST version 1.1, ypT and ypN downstaging, R0 resection, immune-related adverse events, grade 3 or higher treatment-related adverse events, postoperative complications graded by the Clavien–Dindo classification, recurrence within 12 months, and recurrence-free survival (RFS), defined as the interval from treatment initiation to documented recurrence or death from any cause. Patients without an event were censored at the date of last follow-up.

### Machine-learning benchmarking

Ten commonly used supervised algorithms were compared for MPR prediction using the five-marker panel: logistic regression with L2 regularization, LASSO (L1) logistic regression, elastic-net logistic regression, random forest (31), support vector machine with a radial-basis kernel (32), k-nearest neighbors, a single decision tree, gradient-boosted trees (XGBoost) (33), LightGBM (34), and AdaBoost. This comparison was intended as an exploratory benchmarking exercise rather than an exhaustively optimized contest between learners. All models were fitted within the training cohort only, hyperparameters were tuned by the same five-fold cross-validation on the training data, and the external validation cohort was never used for model fitting or tuning; default library hyperparameter ranges were used for the tree-based learners. Discrimination was summarized by the area under the receiver operating characteristic curve (AUC) with 1000-sample bootstrap 95% confidence intervals (CIs), accuracy, sensitivity, specificity, F1 score, and Brier score in each cohort, together with five-fold cross-validated AUC in the training cohort. Because the panel is low-dimensional and the primary goal was an interpretable, well-calibrated, externally transportable score, the algorithm with the best external-validation discrimination and the smallest training-to-external gap, together with adequate calibration and full interpretability, was carried forward as the ENI-Score backbone.

### Model interpretation, incremental value, and clinical utility

Marker-level contributions to the backbone model were quantified by Shapley additive explanations (SHAP), reporting both the mean absolute SHAP value as a global importance measure and the signed SHAP distribution to indicate the direction of each effect (35). Univariable and multivariable logistic regression evaluated the ENI-Score as a predictor of MPR, with the multivariable model adjusting for age, sex, Eastern Cooperative Oncology Group performance status, cT4 stage, cN2/N3 stage, gastroesophageal junction origin, PD-L1 CPS ≥5, MMR/MSI status, and chemotherapy regimen; estimates are reported as odds ratios (OR) with 95% CIs. The incremental value of the ENI-Score over tumor-centered staging was assessed by comparing two nested logistic models, a clinical-staging baseline and the baseline plus ENI-Score, using the DeLong test for correlated AUCs (36). Calibration was assessed by calibration plots together with the calibration slope, calibration intercept, and Brier score in each cohort, and by the Hosmer–Lemeshow goodness-of-fit test; clinical utility was assessed by decision-curve analysis across a range of threshold probabilities (37). Robustness of the five-marker panel was further examined through a Spearman correlation matrix and variance inflation factors among the markers, drop-one-marker sensitivity analyses, and domain-restricted sub-panels (**Supplementary Tables 3**, **S4**). The independence of the ENI-Score as a prognostic factor was tested in sequential Cox models that progressively adjusted for MPR and other pathological response variables and in models stratified by MPR status (**Supplementary Table 5**).

### Statistical analysis

Continuous variables are summarized as median (interquartile range, IQR) and categorical variables as n (%). Two-group comparisons used the Mann–Whitney U test for continuous variables and the χ² or Fisher exact test for categorical variables; three-cohort comparisons used the Kruskal–Wallis test, and trends across ordered ENI-Score groups used the Cochran–Armitage test. Survival was estimated by the Kaplan–Meier method and compared by the log-rank test, with adjusted hazard ratios from Cox proportional-hazards models. Analyses were performed in Python 3.11 (scikit-learn, statsmodels, lifelines, xgboost, lightgbm, and shap), and figures were reproduced in R 4.4.0 (pROC, rms, survminer, and ggplot2). All tests were two-sided, and P<0.05 was considered statistically significant. With 738 patients and an event (MPR) fraction of approximately 40%, the effective sample supported the development and three-way validation of a five-variable model while maintaining well over ten events per candidate predictor.

## Results

### Patient characteristics

Of 738 eligible patients, 335 formed the training cohort, 114 the internal validation cohort, and 289 the external validation cohort. The median age was 62 years (IQR 56-67), and 490 patients (66.4%) were men. Gastroesophageal junction or upper-third tumors accounted for 96 cases (13.0%); 352 patients (47.7%) had cT4 disease and 397 (53.8%) had cN2/cN3 disease. PD-L1 CPS reached 5 or higher in 442 patients (59.9%), and 54 (7.3%) had dMMR/MSI-H tumors. The most frequent chemotherapy backbone was SOX (337 patients), followed by XELOX (200), FLOT (137), and DOS (64), and the median number of preoperative cycles was four. Overall, 302 patients (40.9%) achieved MPR, 112 (15.2%) pCR, 455 (61.7%) an objective response, and 684 (92.7%) an R0 resection. Demographic, tumor, treatment, and laboratory characteristics, including the MPR rate, were well balanced across the three cohorts (MPR P = 0.96; all other P>0.05 except a minor difference in dMMR prevalence), supporting their use for independent validation, as detailed in **Table 1**.

### Endocrine-nutritional markers differ sharply by MPR status

**Figure 2:**
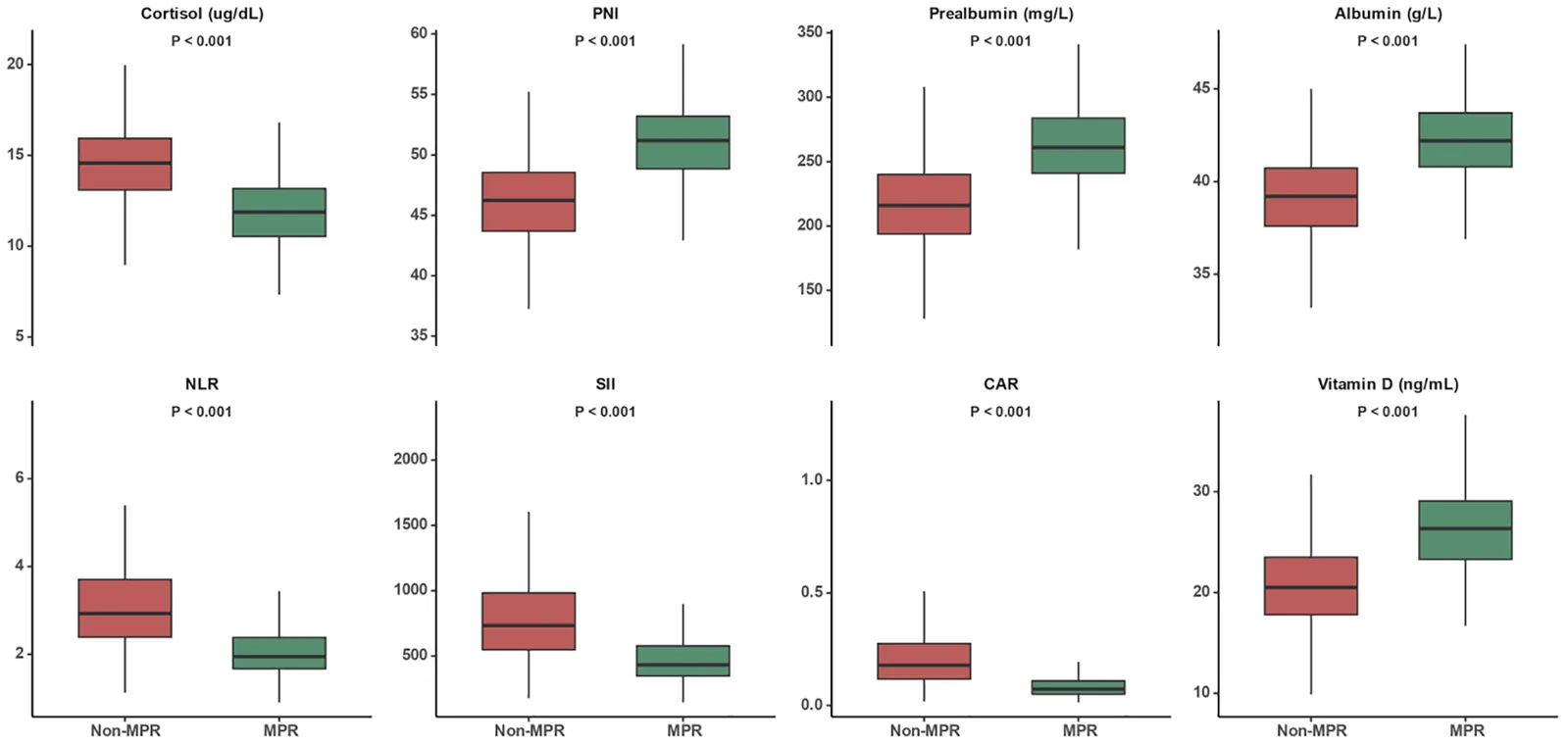
Pretreatment host-state markers by MPR status (n=738). Box plots compare MPR and non-MPR patients for cortisol, PNI, prealbumin, albumin, NLR, SII, CAR, and vitamin D, with Mann–Whitney P values.

Across the full cohort of 738 patients, every component of the ENI panel and most related host-state markers separated MPR from non-MPR patients with a large and biologically coherent difference (each P<0.001 for the five panel markers). Patients who achieved MPR had lower 8 AM cortisol (median 11.9 vs 14.6 µg/dL), higher PNI (51.2 vs 46.2), higher prealbumin (261 vs 216 mg/L), higher albumin (42.2 vs 39.2 g/L), lower NLR (1.94 vs 2.92), lower CAR (0.07 vs 0.18), lower CRP (3.1 vs 7.0 mg/L), lower SII (432 vs 734), and higher 25-hydroxy vitamin D (26.4 vs 20.5 ng/mL). Related markers moved consistently, with higher lymphocyte count, higher lymphocyte-to-monocyte ratio, and higher hemoglobin in responders and higher neutrophil, monocyte, platelet, and ferritin in non-responders, whereas most purely metabolic markers did not differ. Critically, the direction of every significant difference was preserved across the training, internal validation, and external validation cohorts. Marker distributions by MPR status are summarized in **Table 2** and displayed in **Figure 2**.

**Table 2:** Pretreatment host-state markers by MPR status (full cohort, n=738).

Marker	Domain	MPR (n=302)	Non-MPR (n=436)	P
Cortisol, µg/dL	Endocrine	11.88 (10.54-13.17)	14.57 (13.10-15.93)	<0.001
FT3, pmol/L	Endocrine	4.64 (4.44-4.88)	4.28 (4.03-4.48)	<0.001
Albumin, g/L	Nutrition	42.20 (40.80-43.70)	39.20 (37.60-40.73)	<0.001
Prealbumin, mg/L	Nutrition	261 (241-284)	216 (194-240)	<0.001
PNI	Nutrition	51.18 (48.85-53.19)	46.23 (43.69-48.54)	<0.001
Vitamin D, ng/mL	Micronutrient	26.35 (23.30-29.08)	20.50 (17.80-23.50)	<0.001
Lymphocyte, 10^9^/L	Inflammation	1.75 (1.59-1.93)	1.42 (1.23-1.59)	<0.001
Neutrophil, 10^9^/L	Inflammation	3.47 (3.13-3.91)	4.16 (3.79-4.59)	<0.001
CRP, mg/L	Inflammation	3.09 (2.20-4.47)	6.96 (4.73-10.30)	<0.001
NLR	Inflammation	1.94 (1.67-2.38)	2.92 (2.39-3.70)	<0.001
SII	Inflammation	432 (347-577)	734 (548-983)	<0.001
CAR	Inflammation	0.07 (0.05-0.11)	0.18 (0.12-0.28)	<0.001
ENI-Score	Score	64.55 (40.17-80.40)	17.45 (6.92-35.60)	<0.001

Values are median (IQR); P values are from Mann–Whitney U tests. The direction of every difference was consistent across all three cohorts. FT3, free triiodothyronine; SII, systemic immune-inflammation index; other abbreviations as in **Table 1**.

### Logistic regression gives the best external AUC among ten algorithms

**Figure 3:**
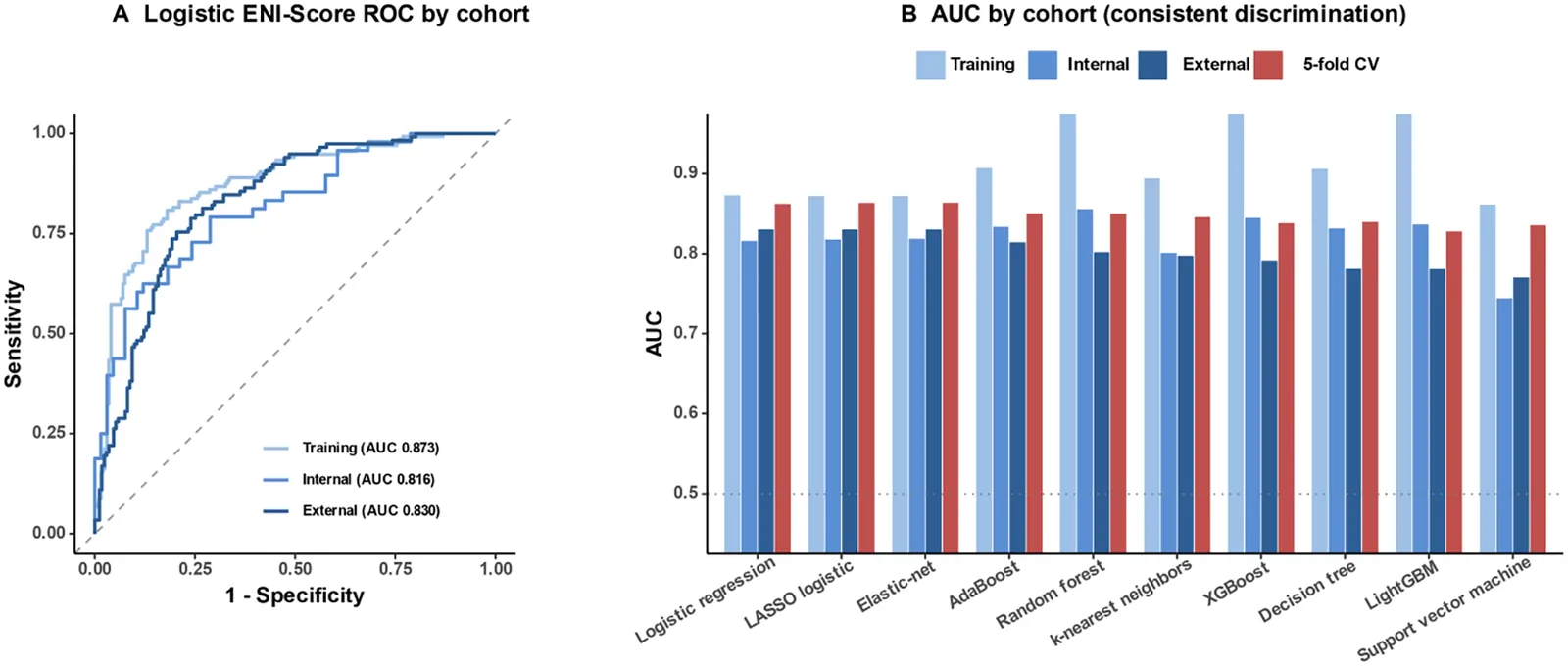
Discrimination of ten machine-learning models: **(A)** logistic ENI-Score ROC curves across the training, internal, and external cohorts; **(B)** training, internal, external, and cross-validated AUC by model.

**Figure 4:**
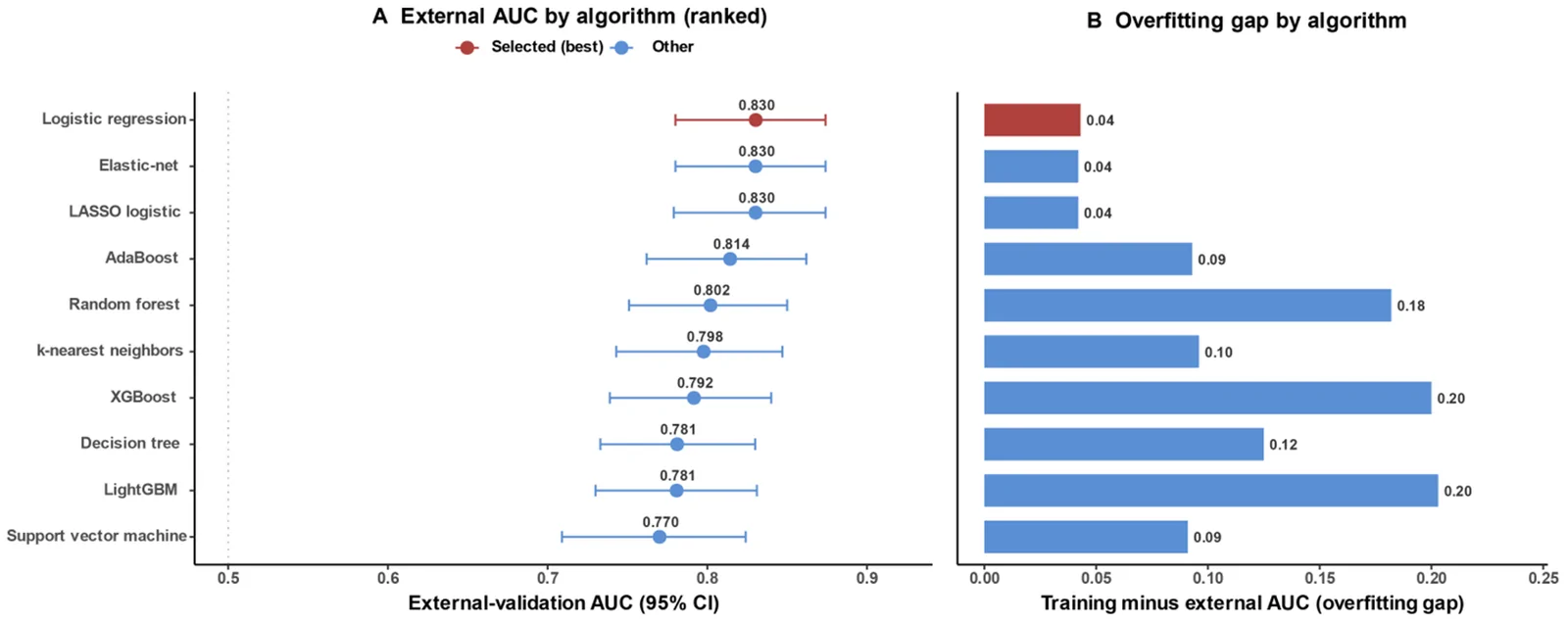
Comparison of ten machine-learning algorithms: (A) external-validation AUC with 95% bootstrap confidence intervals; (B) training-to-external AUC gap, with algorithms ranked by external-validation AUC. Regularized logistic regression, LASSO logistic regression, and elastic-net achieved the joint-highest external-validation AUC, whereas tree-based ensembles showed the largest overfitting gaps.

Ten commonly used machine-learning algorithms were compared in 738 patients. Regularized logistic regression achieved the highest external-validation AUC (0.830, 95% CI 0.78-0.87), with its penalized variants LASSO and elastic-net essentially identical (both 0.830) and a five-fold cross-validated AUC of 0.862. The remaining algorithms generalized less well, in descending external AUC: AdaBoost (0.814), random forest (0.802), k-nearest neighbors (0.798), XGBoost (0.792), decision tree and LightGBM (both 0.781), and support vector machine (0.770). Tree-based ensembles reached near-perfect training AUC (0.98 to 0.99) but lost 0.18 to 0.20 of AUC on external data, a clear pattern of overfitting on the compact five-marker feature space, whereas the logistic model showed the smallest training-to-external gap (0.04) and the most stable behavior across cohorts. Given this combination of accuracy, stability, calibration, and interpretability, regularized logistic regression was selected as the ENI-Score backbone. Full performance across cohorts is reported in **Table 3** and **Figure 3**, and the external-AUC ranking of all ten algorithms is shown in **Figure 4**.

**Table 3:** Discrimination of ten machine-learning algorithms for MPR.

Model	Training AUC	Internal AUC	External AUC	5-fold CV AUC	External brier
Logistic regression	0.873	0.816	0.830	0.862	0.170
LASSO logistic	0.872	0.818	0.830	0.864	0.169
Elastic-net	0.872	0.819	0.830	0.864	0.170
AdaBoost	0.907	0.834	0.814	0.850	0.197
Random forest	0.984	0.856	0.802	0.850	0.185
k-nearest neighbors	0.894	0.801	0.798	0.846	0.185
XGBoost	0.991	0.845	0.792	0.838	0.200
Decision tree	0.906	0.832	0.781	0.840	0.204
LightGBM	0.984	0.837	0.781	0.828	0.206
Support vector machine	0.861	0.744	0.770	0.836	0.188

AUC, area under the ROC curve; CV, cross-validation. Models are ordered by external-validation AUC. Regularized logistic regression achieved the highest external AUC and was selected as the ENI-Score backbone; tree-based ensembles reached near-perfect training AUC but lost substantial AUC on external data, indicating overfitting on the compact five-marker space.

### Calibration and clinical net benefit

**Figure 5:**
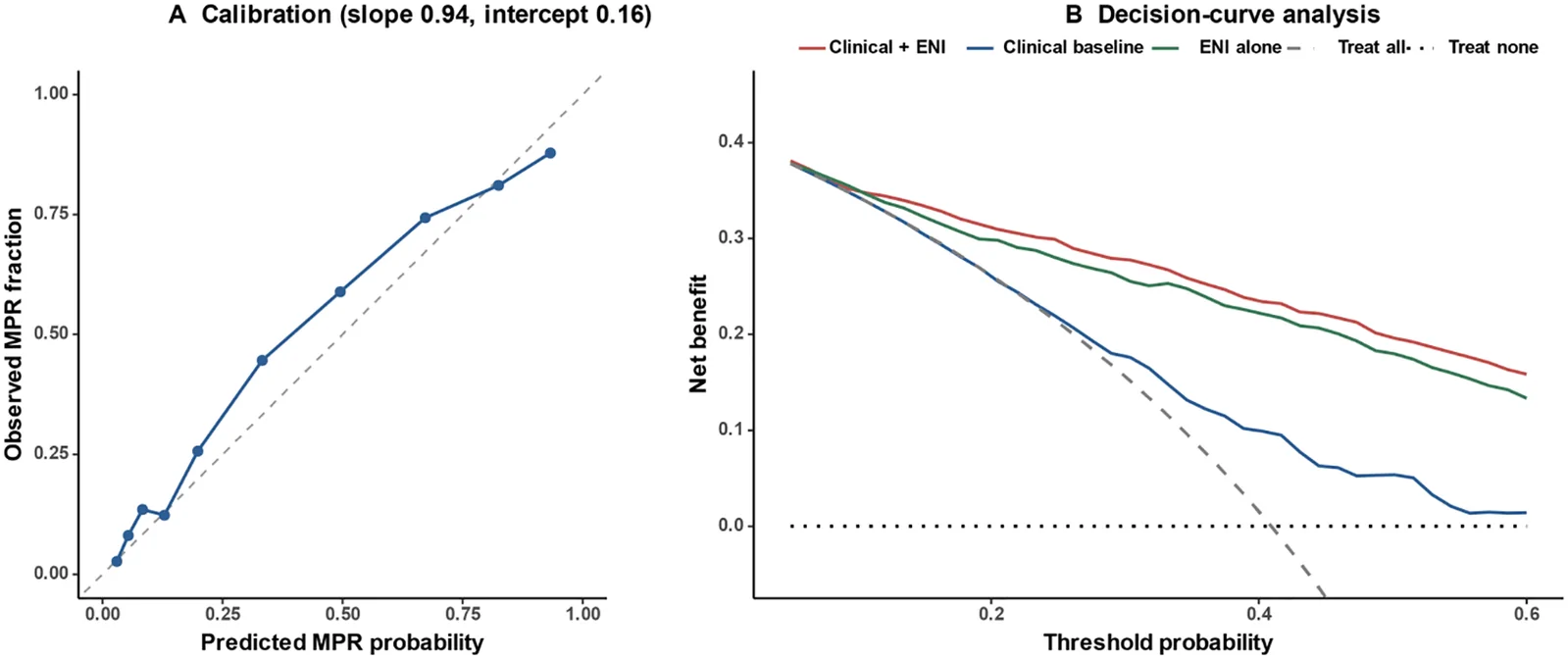
Calibration and clinical utility of the ENI-Score: **(A)** calibration plot comparing observed and predicted probabilities of MPR; **(B)** decision-curve analysis comparing the clinical-plus-ENI model, clinical baseline model, ENI-Score alone, treat-all strategy, and treat-none strategy.

Calibration plots showed generally acceptable agreement between predicted and observed MPR probabilities across the full score range. The overall calibration slope was 0.94 and the calibration intercept 0.16, with per-cohort slopes of 1.04 (training), 0.87 (internal), and 0.90 (external) and Brier scores of 0.14 to 0.17 (**Supplementary Table 6**; **Supplementary Figure 1**). The Hosmer–Lemeshow test was statistically significant (P = 0.026), indicating a small but detectable deviation from perfect calibration that most likely reflects the large sample size rather than clinically meaningful miscalibration. Decision-curve analysis demonstrated a clear positive net benefit relative to the treat-all and treat-none strategies across a wide band of threshold probabilities, and the ENI-Score and the combined clinical-plus-ENI model both exceeded the clinical-staging baseline across clinically relevant thresholds. Calibration and decision curves are shown in **Figure 5**.

### External validation is consistent across the three centers

Because the external cohort combined three hospitals, we also examined each center separately (**Supplementary Table 7**; **Supplementary Figure 2**). Discrimination was consistent, with center-specific AUCs of 0.875 (n=97), 0.815 (n=96), and 0.788 (n=96); MPR rates ranged from 35.4% to 44.3%; calibration slopes ranged from 0.68 to 1.15; and Brier scores ranged from 0.145 to 0.184. The modestly lower AUC at the third center, which also had the lowest MPR rate, is most plausibly explained by its smaller size and differences in case mix and assay platforms, and the overall pattern supports stable transportability rather than a result driven by a single site. Discrimination remained clinically acceptable at every center (all center-specific areas under the curve at or above 0.79, with overlapping confidence intervals and calibration slopes spanning 0.68 to 1.15), so the claim of multicenter generalizability does not rest on any single site. The MPR gradient and discrimination of the ENI-Score were likewise preserved across chemotherapy backbones and cycle numbers (**Supplementary Table 8**): the pooled area under the curve was 0.844 in patients receiving an oxaliplatin doublet (SOX or XELOX, n=537) and 0.861 in those receiving a docetaxel triplet (FLOT or DOS, n=201), and 0.807 versus 0.861 in patients receiving three or fewer versus four or more preoperative cycles, with a monotonic Low-to-High MPR increase in every subgroup.

### The five markers are correlated and the panel is robust but parsimonious

Because the panel mixes nutritional and inflammatory indices that share constituent laboratory values, we explicitly examined redundancy (**Supplementary Tables 3, S4**). The five markers were strongly inter-correlated (absolute Spearman 0.89 to 0.95; **Supplementary Figure 3**) and showed elevated variance inflation factors (PNI 12.4, NLR 8.4, prealbumin 7.5, cortisol 6.6, CAR 4.0), confirming substantial overlap. In drop-one-marker analyses the external AUC remained essentially unchanged when any single marker was removed (0.824 to 0.831), and domain-restricted sub-panels performed similarly to the full panel (endocrine-plus-nutritional three-marker external AUC 0.831; inflammatory two-marker 0.830; full five-marker 0.830; **Supplementary Figure 4**). These results indicate that no single marker is independently indispensable and that smaller sub-panels capture much of the signal; the five-marker panel was therefore retained for its balanced representation of the three host-state domains and its interpretability rather than because each marker contributes statistically independent information, a point we make explicit to avoid overstating marker independence. To benchmark the composite score against the simplest routine alternatives, we compared the discrimination of each individual marker with that of the full ENI-Score in the pooled cohort (**Supplementary Table 9**): PNI alone reached an area under the curve of 0.840, CAR 0.839, cortisol 0.829, prealbumin 0.828, and NLR 0.826, whereas the full five-marker ENI-Score reached 0.848 and clinical staging plus the ENI-Score reached 0.857. The ENI-Score significantly outperformed cortisol (DeLong P = 0.005) and NLR (P<0.001) alone, whereas its advantage over PNI alone was small and non-significant (P = 0.12), indicating that most discrimination is carried by the nutritional axis while the additional markers contribute interpretability and robustness rather than large incremental discrimination.

### SHAP analysis ranks nutritional and endocrine markers highest

**Figure 6:**
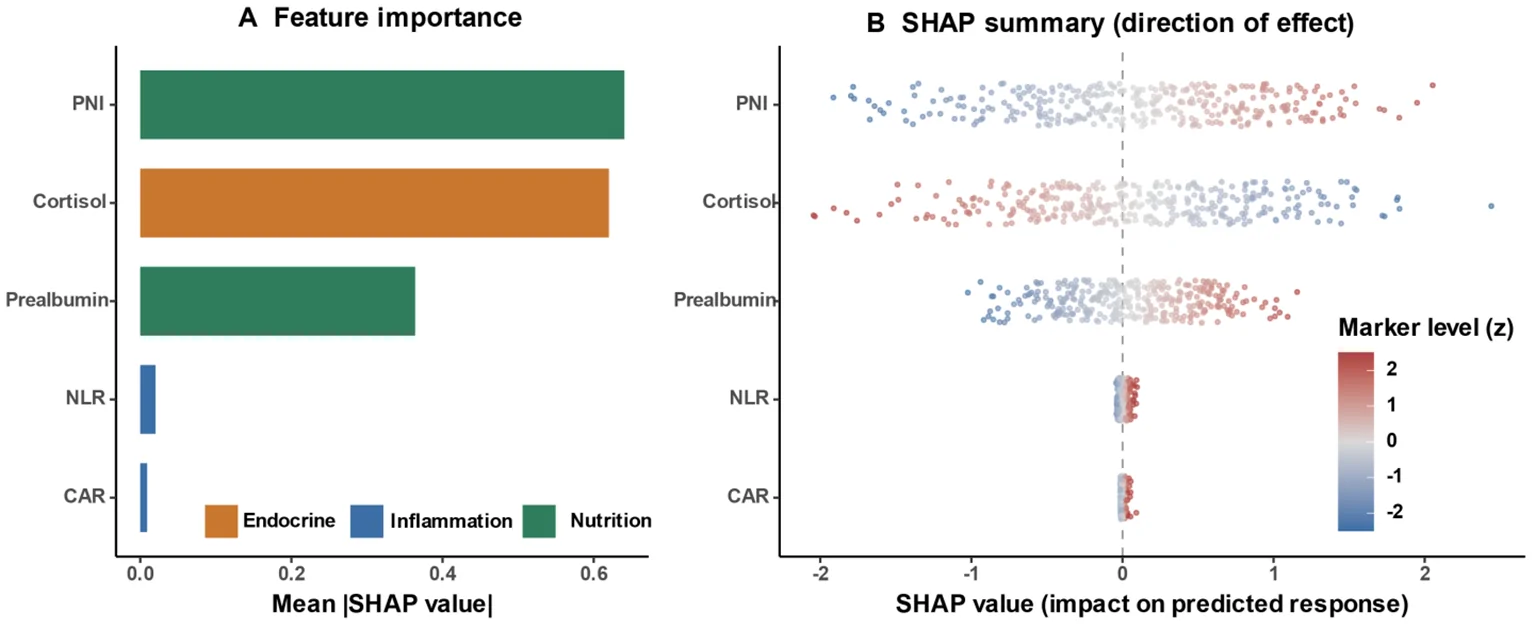
SHAP interpretability of the ENI-Score: **(A)** mean absolute SHAP value for each marker; **(B)** SHAP summary beeswarm plot showing the direction and magnitude of each marker’s effect on the predicted probability of MPR.

In the training cohort of 335 patients, SHAP analysis ranked PNI as the most influential marker (mean absolute SHAP 0.64), followed closely by 8 AM cortisol (0.62) and prealbumin (0.36), with smaller independent contributions from NLR and CAR once the nutritional and endocrine markers were accounted for, consistent with partial correlation among the inflammatory indices. The signed SHAP distribution showed that higher cortisol, higher NLR, and higher CAR shifted predictions toward non-response, whereas higher PNI and higher prealbumin shifted predictions toward response, mirroring the univariable marker comparisons and supporting the biological coherence of the model. The SHAP summary and contribution plots are shown in **Figure 6**.

### ENI-score tertiles stratify response and augment clinical staging

**Figure 7:**
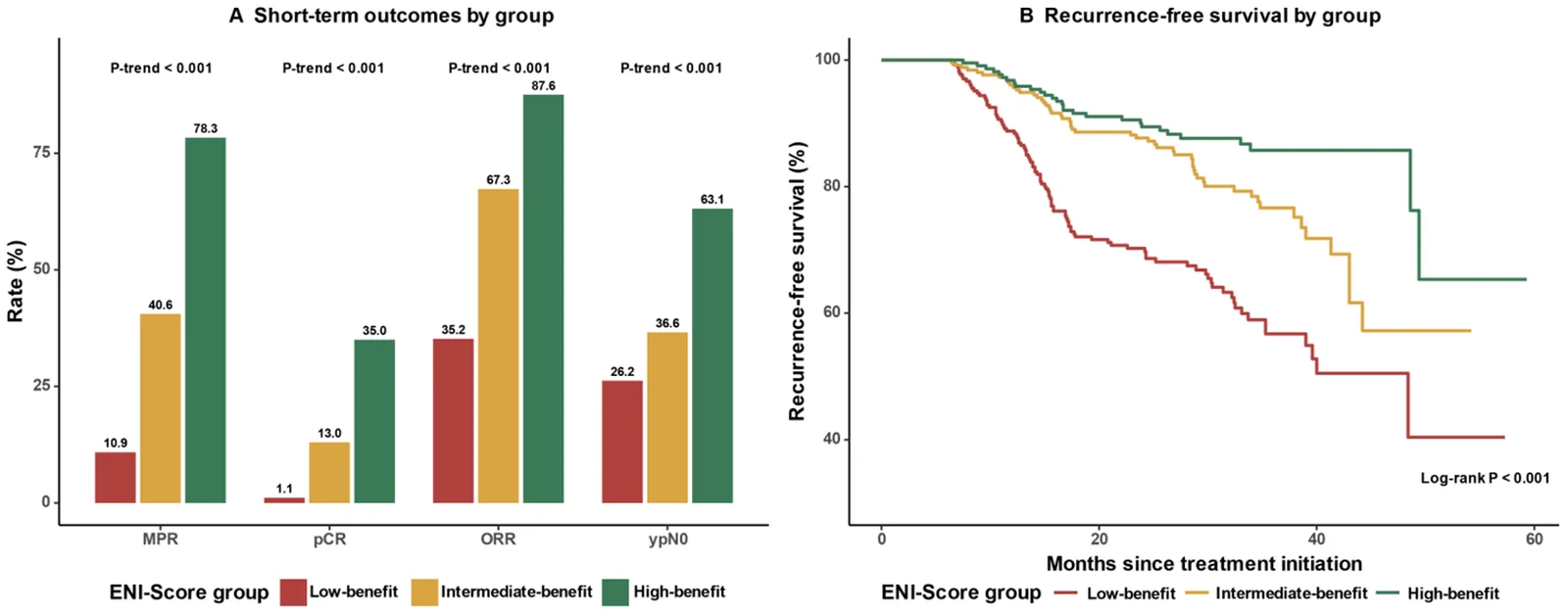
Clinical stratification by the ENI-Score: **(A)** rates of MPR, pCR, ORR, and ypN0 across the Low-, Intermediate-, and High-benefit groups; **(B)** Kaplan–Meier recurrence-free survival curves for the three ENI-Score groups. The between-group difference was significant by the log-rank test (P<0.001).

Applying the fixed training-derived cutoffs classified 251 patients as Low-benefit, 267 as Intermediate-benefit, and 220 as High-benefit. MPR rates rose steeply across these groups (10.4%, 38.2%, and 79.1%; P-trend<0.001), as did pCR (1.2%, 12.0%, 35.0%), ORR (33.5%, 66.7%, 87.7%), and ypN0 downstaging (25.1%, 37.1%, 62.7%). After adjustment for age, sex, performance status, cT4, cN2/N3, gastroesophageal junction origin, PD-L1 CPS ≥5, MMR/MSI status, and regimen, the odds ratio for MPR was 35.3 (95% CI 20.6-60.5) for High-benefit versus Low-benefit and 5.5 (95% CI 3.4-8.9) for Intermediate-benefit versus Low-benefit, and per 10-point increase the adjusted OR was 1.72 (95% CI 1.59-1.86, P<0.001). In the same multivariable model the ENI-Score, PD-L1 CPS ≥5 (OR 2.15, 95% CI 1.44-3.21), and dMMR/MSI-H (OR 3.70, 95% CI 1.79-7.65) were each independent predictors of MPR, indicating complementary host-side and tumor-side information. The ENI-Score was essentially uncorrelated with PD-L1 combined positive score (Spearman rho 0.01) and only marginally higher in dMMR/MSI-H tumors, and it retained strong, independent discrimination within tumor-biomarker strata (**Supplementary Table 10**): the area under the curve was 0.835 in patients with PD-L1 CPS of 5 or higher and 0.880 in those with CPS below 5, and 0.852 in the pMMR/MSS subgroup, with a significant per 10-point odds ratio in every stratum, so the host-state score conveys information largely orthogonal to established tumor-side biomarkers. Because the tertile boundaries are operational rather than clinical thresholds, we also report their diagnostic characteristics for MPR (**Supplementary Table 11**): the lower cut-point (score 42.4) achieved 91.4% sensitivity, 51.6% specificity, and 89.6% negative predictive value and functions as a rule-out boundary, whereas the upper cut-point (score 72.9) achieved 89.4% specificity and 79.1% positive predictive value and functions as a rule-in boundary. The host-only ENI-Score reached an overall AUC of 0.842; a clinical-staging baseline reached 0.656, and adding the ENI-Score raised the overall AUC to 0.864, a significant improvement by the DeLong test (P<0.001). Beyond the primary endpoint, the ENI-Score also discriminated secondary outcomes in the pooled cohort (**Supplementary Table 12**), with areas under the curve of 0.823 for pCR, 0.780 for objective response, 0.680 for ypN0 downstaging, and 0.669 for recurrence within 12 months. Group outcomes are reported in **Table 4** and **Table 5**, and the proposed clinical pathway is shown in **Figure 7**.

**Table 4:** Univariable and multivariable logistic regression for MPR (n=738).

Variable	Univariable OR (95% CI)	P	Multivariable OR (95% CI)	P
ENI-Score (per 10 points)	1.67 (1.56-1.80)	<0.001	1.72 (1.59-1.86)	<0.001
Age (per year)	0.99 (0.97-1.00)	0.142	1.00 (0.98-1.02)	0.821
Male sex	0.77 (0.56-1.05)	0.096	0.83 (0.56-1.25)	0.373
ECOG PS ≥1	0.69 (0.51-0.93)	0.016	0.72 (0.49-1.07)	0.101
Clinical T4	0.57 (0.42-0.76)	<0.001	0.52 (0.35-0.77)	<0.001
Clinical N2/N3	0.64 (0.48-0.87)	0.004	0.62 (0.42-0.92)	0.017
EGJ origin	1.09 (0.71-1.68)	0.703	1.32 (0.75-2.31)	0.338
PD-L1 CPS ≥5	1.77 (1.31-2.41)	<0.001	2.15 (1.44-3.21)	<0.001
dMMR/MSI-H	3.14 (1.75-5.65)	<0.001	3.70 (1.79-7.65)	<0.001
XELOX vs SOX	1.06 (0.76-1.48)	0.716	1.85 (1.15-2.98)	0.011
FLOT vs SOX	1.19 (0.80-1.77)	0.394	1.28 (0.76-2.16)	0.358
DOS vs SOX	1.14 (0.66-1.97)	0.641	1.26 (0.61-2.61)	0.531

OR, odds ratio; CI, confidence interval. The multivariable model mutually adjusts for all listed variables; the ENI-Score, PD-L1 CPS ≥5, and dMMR/MSI-H remain independent predictors. Abbreviations as in **Table 1**.

**Table 5:** Short-term outcomes and early recurrence by ENI-Score group.

Outcome	Low-benefit (n=251)	Intermediate (n=267)	High-benefit (n=220)	P-trend
MPR	26 (10.4)	102 (38.2)	174 (79.1)	<0.001
pCR	3 (1.2)	32 (12.0)	77 (35.0)	<0.001
ORR	84 (33.5)	178 (66.7)	193 (87.7)	<0.001
DCR	210 (83.7)	252 (94.4)	218 (99.1)	<0.001
ypN0	63 (25.1)	99 (37.1)	138 (62.7)	<0.001
R0 resection	224 (89.2)	246 (92.1)	214 (97.3)	0.001
Any irAE	61 (24.3)	74 (27.7)	60 (27.3)	0.887
Recurrence ≤12 mo	59 (23.5)	26 (9.7)	18 (8.2)	<0.001

Values are n (%). P-trend is from the Cochran–Armitage trend test across ordered ENI-Score groups. irAE, immune-related adverse event; other abbreviations as in **Table 1**.

### ENI-score is associated with recurrence-free survival

At a median follow-up of 28.8 months, recurrence within 12 months fell monotonically across ENI-Score groups (23.5%, 9.7%, and 8.2%; P-trend<0.001). Recurrence-free survival differed significantly between the three groups (log-rank P<0.001), with the Low-benefit group showing the steepest early decline; per 10-point increase in ENI-Score the adjusted hazard ratio (HR) for recurrence was 0.78 after adjustment for clinical factors (P<0.001). Because the ENI-Score predicts MPR and MPR itself influences recurrence, we tested whether the prognostic signal was independent of pathological response in sequential Cox models (**Supplementary Table 5**; **Supplementary Figure 5**). The association persisted after further adjustment for MPR (HR per 10 points 0.81, P<0.001) and for pCR, ypN0, and R0 resection (HR 0.81, P<0.001), and remained significant within both the MPR (HR 0.82, P = 0.006) and non-MPR (HR 0.81, P<0.001) strata. This indicates that the ENI-Score carries prognostic information for early recurrence that is not fully mediated by pathological response. Thus, beyond predicting short-term pathological response, a higher ENI-Score identified patients at lower early recurrence risk, although the moderate follow-up duration means these survival findings should be regarded as early signals. In a sensitivity analysis restricted to 12-month recurrence, each 10-point increase in the ENI-Score carried an adjusted odds ratio of 0.78 (95% CI 0.72 to 0.86, P<0.001), and the association with recurrence-free survival persisted in a multivariable Cox model additionally adjusted for MPR, pCR, ypN0, and R0 resection (adjusted HR per 10 points 0.81, 95% CI 0.75 to 0.87; **Supplementary Table 13**). Given the moderate follow-up and the study’s primary focus on short-term pathological response, these survival findings are presented as exploratory and hypothesis-generating. Kaplan–Meier curves by ENI-Score group are shown in **Figure 7**.

### Safety and surgical outcomes by ENI-score group

Safety and surgical endpoints were tabulated by ENI-Score group (**Supplementary Table 14**). The rate of grade 3 or higher treatment-related adverse events was similar across Low-, Intermediate-, and High-benefit groups (10.8%, 13.5%, and 14.1%), as was any immune-related adverse event (24.3%, 27.7%, and 27.3%). In contrast, postoperative complications (22.7%, 17.6%, and 14.5%) and Clavien–Dindo grade III or higher complications (5.6%, 4.9%, and 2.7%) decreased as the ENI-Score increased, consistent with the better nutritional reserve of higher-scoring patients. Thus a higher ENI-Score was not associated with greater toxicity and was accompanied by fewer severe surgical complications. In a logistic model adjusting for age, sex, body mass index, cT4, cN2/N3, and diabetes, each 10-point increase in the ENI-Score remained independently associated with fewer postoperative complications (adjusted odds ratio 0.92, 95% CI 0.85 to 0.99, P = 0.022; **Supplementary Table 15**), consistent with the score also indexing perioperative physiological reserve.

## Discussion

In this multicenter study of 738 patients with locally advanced gastric or gastroesophageal junction adenocarcinoma, a five-marker peripheral-blood signature drawn from the endocrine, nutritional, and inflammatory axes strongly and reproducibly predicted major pathological response after neoadjuvant immunochemotherapy. Each component marker differed markedly between MPR and non-MPR patients with a consistent direction across three independent cohorts, and the resulting ENI-Score stratified MPR rates from roughly 11% in the Low-benefit group to 78% in the High-benefit group. The score discriminated well in external validation, significantly augmented tumor-centered clinical staging, and was additionally associated with recurrence-free survival. Because every input is a routine, inexpensive laboratory value and the backbone model is fully interpretable, the ENI-Score is readily reproducible and clinically transparent.

The biological reading of the signature is internally coherent and concordant with established immunology. Endogenous glucocorticoids, indexed here by morning cortisol and ranked near the top of the model by SHAP, are produced within the tumor microenvironment by myeloid cells, transactivate multiple checkpoint receptors, and drive CD8 T-cell dysfunction, and active glucocorticoid signaling has been associated with failure of checkpoint blockade (15, 16). Glucocorticoid receptor activation can also upregulate tumor PD-L1 and promote immune evasion (17), providing a mechanistic basis for the lower cortisol observed in responders. Low PNI and low prealbumin mark depleted nutritional reserve and impaired effector capacity and have predicted immunotherapy outcome in other tumors (18, 19), while elevated NLR and CAR mark a myeloid-skewed, systemically inflamed milieu that is repeatedly associated with inferior checkpoint-inhibitor efficacy (18, 20). The dominance of PNI, cortisol, and prealbumin in the SHAP ranking, with NLR and CAR contributing additional but partly overlapping information, is consistent with the known correlation among circulating inflammatory indices and supports a parsimonious, domain-balanced panel.

A central and clinically important observation is that the host-side ENI-Score and the tumor-side markers were each independent predictors of MPR, and that combining them produced the highest discrimination. PD-L1 CPS ≥5 and dMMR/MSI-H retained significant odds ratios in the multivariable model alongside the ENI-Score, and adding the score to a clinical-staging baseline significantly improved the AUC. This indicates that the host and tumor axes carry complementary rather than redundant information, in keeping with the molecular heterogeneity of gastric cancer and the incomplete response enrichment achieved by tumor-side biomarkers alone (12–14). The finding aligns with a broader body of work, including our own, showing that multimodal integration of orthogonal data sources, such as radiomic, pathomic, and transcriptomic features, improves non-invasive prediction of peritoneal metastasis, lavage-cytology positivity, and postoperative recurrence in gastric cancer (21–28). The ENI-Score adds a low-cost, blood-based host-state dimension that could be combined with such imaging- and tissue-based models in future integrated frameworks.

Several features support potential clinical relevance. Every component is drawn from routine pretreatment workup at essentially no added cost, the markers are reproducible and globally available, and the score is transparent rather than a black box, which facilitates trust and external use. The steep MPR gradient across tertiles, the large adjusted High-versus-Low odds ratio, the significant incremental AUC over staging, and the association with recurrence-free survival together suggest that a pretreatment host-state assessment could help flag patients with a lower probability of benefit who might be considered for closer response monitoring, nutritional and anti-inflammatory optimization, or intensified or alternative strategies, hypotheses that are directly testable in prospective trials. Importantly, the score is intended to complement, not replace, tumor-side staging and biomarkers. At the current stage of evidence, the ENI-Score should be regarded as a pretreatment risk-stratification and monitoring tool rather than a treatment-decision rule: it is not yet suitable for selecting, intensifying, escalating, or de-escalating neoadjuvant immunochemotherapy, and prospective validation will be required before any such treatment-modifying use.

Our methodological approach also carries a practical lesson. Across ten algorithms, the regularized logistic model achieved the best external-validation AUC and the smallest overfitting gap, whereas flexible tree ensembles attained near-perfect training discrimination that did not transport. On a compact, low-dimensional panel with a few hundred training events, a parsimonious, well-calibrated linear model can match or exceed more complex learners while remaining interpretable, an outcome consistent with prudent model selection in clinical prediction. This argues against reflexively adopting the most flexible algorithm and in favor of transparent models supported by formal calibration and decision-curve evaluation.

This study has limitations. It is retrospective, and although cohorts were separated by center and time, residual confounding and selection effects cannot be excluded. Most importantly, because pathological response can only be assessed in patients who complete neoadjuvant treatment and undergo surgery, the ENI-Score should be interpreted as predicting MPR among surgical completers rather than treatment benefit in the full intention-to-treat population; patients who progressed, developed prohibitive toxicity, or did not reach surgery were necessarily excluded, which may inflate apparent performance and limits extrapolation to the moment of treatment selection. Because a complete pretreatment laboratory panel was an eligibility criterion, individual-level data were not available for patients excluded before analysis, so a formal comparison of baseline characteristics between included and excluded patients could not be performed and the direction of any resulting selection effect remains uncertain; this should be resolved in a prospective, intention-to-treat evaluation. In addition, the five markers are strongly inter-correlated with elevated variance inflation factors, and sensitivity analyses show that smaller sub-panels perform comparably; the panel should therefore be viewed as a parsimonious, domain-balanced summary of a shared host-state axis rather than as five independently necessary predictors. A single morning cortisol measurement is sensitive to sampling time and acute physiological stress and is an imperfect index of glucocorticoid tone; serial or diurnal sampling might strengthen the endocrine component. Peripheral-blood markers are a distal readout of the tumor immune microenvironment and cannot fully capture it, which sets an intrinsic ceiling on discrimination, and PD-1 agents and chemotherapy backbones were heterogeneous and pooled. The follow-up was of moderate duration, so the survival findings are best interpreted as early signals pending longer observation. Finally, although the cohorts were drawn from four hospitals, all were Chinese, and prospective, multiethnic, biomarker-paired validation will be required before clinical application. Nonetheless, the consistency of marker direction across cohorts, the rigorous three-way validation, the formal calibration and decision-curve analyses, and the transparent algorithm support the robustness of the central findings.

## Conclusions

The ENI-Score integrates five low-cost routine peripheral-blood markers into an interpretable signature that, in retrospective multicenter validation, accurately and reproducibly stratifies major pathological response after neoadjuvant immunochemotherapy in locally advanced gastric cancer, significantly augments tumor-centered clinical staging, and is associated with recurrence-free survival. By indexing the endocrine, nutritional, and inflammatory host environment that conditions checkpoint-inhibitor efficacy, the score provides an inexpensive, transparent adjunct to existing tumor-side biomarkers and a candidate component for future multimodal frameworks. Prospective, biomarker-paired validation is warranted before clinical adoption.

## Data Availability

The original contributions presented in the study are included in the article/**Supplementary Material**. Further inquiries can be directed to the corresponding author.
