# Supplementary material for: Peripheral endocrine–nutritional machine learning signature predicts short-term response to neoadjuvant immunochemotherapy in locally advanced gastric cancer
Source: Front Immunol. 2026 Aug 12;17:1907116. doi: 10.3389/fimmu.2026.1907116 (PMC13506708; doi:10.3389/fimmu.2026.1907116)
Supplement: Supplementary file 1 [file Table1.docx]

# **Supplementary Tables**

**Table S1. Candidate pretreatment host-state markers and rationale for inclusion in the ENI-Score panel**

| **Marker** | **Domain** | **Status** | **Rationale** |
| --- | --- | --- | --- |
| 8 AM cortisol | Endocrine | Included | Peripheral surrogate of glucocorticoid tone; mechanistic link to CD8 T-cell dysfunction and checkpoint resistance |
| PNI | Nutrition | Included | Integrates albumin and lymphocytes; established prognostic nutritional index reflecting effector reserve |
| Prealbumin | Nutrition | Included | Short-half-life marker of acute nutritional status; complements PNI |
| NLR | Inflammation | Included | Indexes myeloid-skewed systemic inflammation; widely validated in immunotherapy |
| CAR | Inflammation | Included | Combines CRP and albumin; robust inflammatory-nutritional ratio |
| Albumin | Nutrition | Not included | Already represented within PNI and CAR; avoids double counting |
| CRP | Inflammation | Not included | Already represented within CAR |
| SII | Inflammation | Not included | Highly correlated with NLR; redundant inflammatory information |
| Lymphocyte / neutrophil | Inflammation | Not included | Constituents of NLR/PNI; captured by composite indices |
| FT3, TSH, FT4 | Endocrine | Not included | Weaker and less specific association; cortisol prioritized for the endocrine axis |
| 25-hydroxy vitamin D | Micronutrient | Not included | Associated with MPR but added little independent value beyond the five-marker panel |
| Ferritin, iron, transferrin | Iron/micronutrient | Not included | Modest, non-independent association; excluded for parsimony |
| Metabolic panel (glucose, lipids) | Metabolic | Not included | No significant association with MPR |

*Markers were prespecified before model training using three criteria: representation of the endocrine, nutritional, and inflammatory axes; routine availability and low cost; and limited redundancy. Abbreviations as in the main text.*

**Table S2. ENI-Score formula: standardized logistic-regression coefficients, training-cohort standardization statistics, and cut-points**

| **Variable** | **Training mean** | **Training SD** | **Coefficient (standardized)** |
| --- | --- | --- | --- |
| 8 AM cortisol (µg/dL) | 13.31 | 2.63 | −0.825 |
| PNI | 48.44 | 4.30 | +0.897 |
| Prealbumin (mg/L) | 237.0 | 41.89 | +0.432 |
| NLR | 2.67 | 1.13 | +0.132 |
| CAR | 0.168 | 0.157 | +0.079 |
| Intercept | — | — | −0.630 |

Each marker is first standardized using the training-cohort mean and SD above: z = (value − mean) / SD. The linear predictor is LP = −0.630 + (−0.825·z_cortisol) + (0.897·z_PNI) + (0.432·z_prealbumin) + (0.132·z_NLR) + (0.079·z_CAR). The predicted MPR probability is p = 1 / (1 + e^(−LP)), and the ENI-Score = 100 × p (range 0–100). Tertile cut-points derived from the training cohort were 19.4 and 57.3, defining Low-benefit (<19.4), Intermediate-benefit (19.4–57.3), and High-benefit (>57.3) groups. Complete marker data were an inclusion criterion, so no imputation is required.Coefficients are on the standardized (z-score) scale. Abbreviations as in the main text.

**Table S3. Inter-marker Spearman correlation and variance inflation factors (training cohort)**

| **Marker** | **Cortisol** | **PNI** | **Prealbumin** | **NLR** | **CAR** | **VIF** |
| --- | --- | --- | --- | --- | --- | --- |
| Cortisol | 1.00 | −0.90 | −0.89 | 0.89 | 0.91 | 6.64 |
| PNI | −0.90 | 1.00 | 0.92 | −0.95 | −0.93 | 12.41 |
| Prealbumin | −0.89 | 0.92 | 1.00 | −0.89 | −0.91 | 7.53 |
| NLR | 0.89 | −0.95 | −0.89 | 1.00 | 0.91 | 8.40 |
| CAR | 0.91 | −0.93 | −0.91 | 0.91 | 1.00 | 4.02 |

*Off-diagonal values are Spearman correlation coefficients; the final column is the variance inflation factor. The high correlations and VIFs confirm substantial redundancy among the five markers.*

**Table S4. Sensitivity analyses: drop-one-marker and domain-restricted sub-panels**

| **Model** | **External AUC** | **Δ vs full panel** |
| --- | --- | --- |
| Full five-marker panel | 0.830 | reference |
| Drop cortisol | 0.831 | +0.001 |
| Drop PNI | 0.824 | −0.006 |
| Drop prealbumin | 0.831 | +0.001 |
| Drop NLR | 0.830 | 0.000 |
| Drop CAR | 0.831 | +0.001 |
| Endocrine + nutritional only (3 markers) | 0.831 | +0.001 |
| Inflammatory only (2 markers) | 0.830 | 0.000 |

*External-validation AUC is essentially unchanged when any single marker is removed, and domain-restricted sub-panels perform comparably, indicating that no single marker is independently indispensable and that the panel is parsimonious.*

**Table S5. Sequential Cox models for recurrence-free survival: ENI-Score per 10 points**

| **Model** | **HR per 10 points (95% CI)** | **P** |
| --- | --- | --- |
| M1: clinical factors | 0.84 (0.79–0.89) | <0.001 |
| M2: M1 + MPR | 0.87 (0.82–0.93) | <0.001 |
| M3: M1 + pCR + ypN0 + R0 | 0.85 (0.80–0.90) | <0.001 |
| Stratified within MPR patients | 0.89 | 0.011 |
| Stratified within non-MPR patients | 0.89 | 0.006 |

*Clinical factors comprise age, sex, cT4, cN2/N3, gastroesophageal junction origin, PD-L1 CPS ≥5, dMMR/MSI-H, and regimen. The ENI-Score remains prognostic after adjustment for pathological response and within response strata, indicating prognostic information not fully mediated by MPR.*

**Table S6. Calibration by cohort**

| **Cohort** | **Calibration slope** | **Calibration intercept** | **Brier score** |
| --- | --- | --- | --- |
| Training | 1.04 | 0.01 | 0.140 |
| Internal validation | 0.87 | 0.33 | 0.173 |
| External validation | 0.90 | 0.24 | 0.170 |
| Overall | 0.94 | 0.16 | 0.157 |

*A slope near 1 and intercept near 0 indicate good calibration; the Hosmer–Lemeshow test was significant overall (P=0.026), reflecting the large sample.*

**Table S7. Center-specific external validation performance**

| **External center** | **n** | **MPR, %** | **AUC (95% CI)** | **Calib. slope** | **Brier** |
| --- | --- | --- | --- | --- | --- |
| Shijiazhuang People's Hospital | 97 | 44.3 | 0.879 (0.814-0.944) | 1.69 | 0.167 |
| Fifth Affil. Hosp. of Anhui Med. Univ. | 96 | 42.7 | 0.822 (0.740-0.903) | 1.38 | 0.188 |
| Yichang Central People's Hospital | 96 | 35.4 | 0.794 (0.698-0.891) | 1.02 | 0.194 |

*Discrimination was clinically acceptable at every center (all AUCs at or above 0.79, overlapping CIs); generalizability does not rest on any single site.*

**Table S8. ENI-Score performance by chemotherapy regimen and cycle number**

| **Subgroup** | **n** | **AUC** | **MPR by tertile Low / Int / High, %** |
| --- | --- | --- | --- |
| SOX | 337 | 0.864 | 6.3 / 37.0 / 76.6 |
| XELOX | 200 | 0.812 | 18.1 / 38.2 / 80.8 |
| FLOT | 137 | 0.828 | 12.5 / 40.4 / 80.0 |
| DOS | 64 | 0.929 | 3.6 / 40.0 / 85.7 |
| Oxaliplatin doublet (SOX or XELOX) | 537 | 0.844 | 10.9 / 37.4 / 78.0 |
| Docetaxel triplet (FLOT or DOS) | 201 | 0.861 | 8.8 / 40.3 / 82.0 |
| Preoperative cycles <= 3 | 179 | 0.807 | 17.3 / 36.6 / 80.4 |
| Preoperative cycles >= 4 | 559 | 0.861 | 8.5 / 38.8 / 78.7 |

*The Low-to-High MPR gradient was monotonic in every subgroup.*

**Table S9. ENI-Score versus single routine markers and clinical staging**

| **Predictor (endpoint MPR, n=738)** | **AUC (95% CI)** | **DeLong vs ENI-Score** |
| --- | --- | --- |
| PNI alone | 0.840 (0.811-0.869) | P=0.12 (NS) |
| C-reactive protein-to-albumin ratio alone | 0.839 (0.810-0.867) | - |
| 8 AM cortisol alone | 0.829 (0.800-0.859) | P=0.005 |
| Prealbumin alone | 0.828 (0.799-0.858) | - |
| Neutrophil-to-lymphocyte ratio alone | 0.826 (0.796-0.856) | P<0.001 |
| ENI-Score (full five-marker panel) | 0.848 (0.820-0.876) | reference |
| Clinical staging + ENI-Score | 0.857 (0.830-0.884) | - |

*AUCs computed on the pooled cohort using the released ENI-Score and markers (cf. cohort-specific values in Table 3). Most discrimination is carried by the nutritional axis; the composite adds interpretability and robustness rather than a large incremental AUC.*

**Table S10. ENI-Score within tumor-biomarker strata**

| **Stratum** | **n** | **MPR, %** | **AUC (95% CI)** | **Per 10-pt OR (95% CI)** |
| --- | --- | --- | --- | --- |
| PD-L1 CPS >= 5 | 442 | 46.4 | 0.835 (0.798-0.872) | 1.81 (1.62-2.03) |
| PD-L1 CPS < 5 | 296 | 32.8 | 0.880 (0.839-0.921) | 2.35 (1.93-2.85) |
| pMMR / MSS | 684 | 38.9 | 0.852 (0.823-0.882) | 1.97 (1.78-2.18) |
| dMMR / MSI-H | 54 | 66.7 | 0.771 (0.614-0.927) | 1.69 (1.22-2.34) |

*ENI-Score was essentially uncorrelated with PD-L1 CPS (Spearman rho 0.01; Mann-Whitney P=0.28) and only marginally higher in dMMR/MSI-H tumors (mean 62.6 vs 54.4; P=0.02). The per 10-point OR was significant in every stratum (all P<0.01), indicating complementary host-side information.*

**Table S11. Diagnostic characteristics at the two tertile cut-points for MPR**

| **Cut-point** | **Sensitivity** | **Specificity** | **PPV** | **NPV** |
| --- | --- | --- | --- | --- |
| Score 42.4 (Low vs Intermediate) | 91.4% | 51.6% | 56.7% | 89.6% |
| Score 72.9 (Intermediate vs High) | 57.6% | 89.4% | 79.1% | 75.3% |

*The lower cut-point behaves as a rule-out boundary (high sensitivity, high NPV); the upper cut-point behaves as a rule-in boundary (high specificity, high PPV).*

**Table S12. ENI-Score discrimination for secondary endpoints**

| **Secondary endpoint** | **Events / n** | **AUC (95% CI)** |
| --- | --- | --- |
| Pathological complete response (pCR) | 112 / 738 | 0.823 (0.787-0.859) |
| Objective response (RECIST) | 455 / 738 | 0.780 (0.746-0.814) |
| ypN0 downstaging | 300 / 738 | 0.680 (0.640-0.720) |
| Recurrence within 12 months | 103 / 738 | 0.669 (0.612-0.725) |

**Table S13. Recurrence-free survival models and 12-month recurrence sensitivity**

| **Model (ENI-Score per 10 points)** | **Estimate (95% CI)** | **P** |
| --- | --- | --- |
| Cox RFS, unadjusted | HR 0.78 (0.73-0.83) | <0.001 |
| Cox RFS + clinical factors | HR 0.78 (0.73-0.83) | <0.001 |
| Cox RFS + clinical + MPR | HR 0.81 (0.76-0.87) | <0.001 |
| Cox RFS + clinical + pathological (MPR, pCR, ypN0, R0) | HR 0.81 (0.75-0.87) | <0.001 |
| Cox RFS within MPR stratum | HR 0.82 (0.71-0.95) | 0.006 |
| Cox RFS within non-MPR stratum | HR 0.81 (0.74-0.88) | <0.001 |
| 12-month recurrence, adjusted logistic | OR 0.78 (0.72-0.86) | <0.001 |

*Clinical factors: age, sex, cT4, cN2/N3, gastroesophageal junction origin. Survival findings are exploratory and hypothesis-generating given the moderate follow-up.*

**Table S14. Safety and surgical outcomes by ENI-Score group**

| **Endpoint** | **Low (n=267)** | **Intermediate (n=254)** | **High (n=217)** | **P-trend** |
| --- | --- | --- | --- | --- |
| Grade ≥3 treatment-related AE | 30 (11.2) | 32 (12.6) | 32 (14.7) | 0.246 |
| Any immune-related AE | 69 (25.8) | 66 (26.0) | 60 (27.6) | 0.640 |
| Postoperative complication | 60 (22.5) | 46 (18.1) | 30 (13.8) | 0.014 |
| Clavien–Dindo ≥III | 15 (5.6) | 12 (4.7) | 6 (2.8) | 0.123 |

*Values are n (%). A higher ENI-Score was not associated with greater toxicity and was accompanied by fewer postoperative complications, consistent with better nutritional reserve. AE, adverse event.*

**Table S15. Adjusted analysis of postoperative complications**

| **Model (ENI-Score per 10 points)** | **OR (95% CI)** | **P** |
| --- | --- | --- |
| Postoperative complication, unadjusted | 0.92 (0.85-0.99) | 0.023 |
| Postoperative complication, adjusted | 0.92 (0.85-0.99) | 0.022 |
| Clavien-Dindo grade III+ , adjusted | 0.91 (0.79-1.05) | 0.19 |

*Adjusted for age, sex, body mass index, cT4, cN2/N3, and diabetes. Complication rates by tertile (Low/Intermediate/High): any complication 22.7 / 17.6 / 14.5%; Clavien-Dindo grade III or higher 5.6 / 4.9 / 2.7%. Findings are consistent with the ENI-Score indexing perioperative physiological reserve.*

# **Supplementary Figure**

**Figure S1.** Inter-marker correlation heatmap. Spearman correlation coefficients among the five ENI panel markers in the training cohort, illustrating the strong inter-marker correlation that motivates the collinearity and sensitivity analyses.

**Figure S2.** Per-center external validation ROC curves. Receiver operating characteristic curves of the ENI-Score in each of the three external centers, with center-specific areas under the curve.

**Figure S3.** Sensitivity analysis of panel composition. External-validation AUC for the full five-marker panel, each drop-one-marker model, and domain-restricted sub-panels, showing that discrimination is preserved across configurations.

**Figure S4.** Calibration by cohort. Calibration curves of predicted versus observed MPR probability in the training, internal validation, and external validation cohorts.

**Figure S5.** Forest plot of recurrence-free survival models. Adjusted hazard ratios (per 10 ENI-Score points) with 95% confidence intervals for the sequential Cox models, showing that the ENI-Score remains prognostic after adjustment for pathological response.
